# Flow Cytometric Challenges in Plasmacytoid Dendritic Cell (pDC) Identification: Limitation of BDCA-4 (CD304)-Based Gating

**DOI:** 10.3390/ijms262210979

**Published:** 2025-11-13

**Authors:** Sarolta Demeter, Tünde Fekete, Beáta Scholtz, Zoltán Veréb, Lajos Kemény, Attila Bácsi, Kitti Pázmándi

**Affiliations:** 1 Department of Immunology, Faculty of Medicine, University of Debrecen, H-4032 Debrecen, Hungary; demeter.sarolta@med.unideb.hu (S.D.); fekete.tunde@med.unideb.hu (T.F.); etele@med.unideb.hu (A.B.); 2 Doctoral School of Molecular Cell and Immune Biology, University of Debrecen, H-4032 Debrecen, Hungary; 3 Department of Biochemistry and Molecular Biology, Faculty of Medicine, University of Debrecen, H-4032 Debrecen, Hungary; scholtz@med.unideb.hu; 4 Regenerative Medicine and Cellular Pharmacology Laboratory, Department of Dermatology and Allergology, Faculty of Medicine, University of Szeged, H-6720 Szeged, Hungary; vereb.zoltan@med.u-szeged.hu (Z.V.); kemeny.lajos@med.u-szeged.hu (L.K.)

**Keywords:** plasmacytoid dendritic cell, monocyte, BDCA-4, gating strategy, flow cytometry

## Abstract

Plasmacytoid dendritic cells (pDCs) are a unique subset of dendritic cells specialized in rapid and robust type I interferon (IFN) production, playing critical roles in the pathogenesis and pathomechanisms of many human diseases. Accurate identification of pDCs in peripheral blood mononuclear cells (PBMCs) is challenging due to dynamic and non-exclusive specific expression of surface markers such as blood dendritic cell antigen (BDCA)-2 and BDCA-4. Although BDCA-4 is generally more stably expressed than BDCA-2, prolonged stimulation or inflammatory conditions can induce its expression on multiple non-pDC cell types, reducing the accuracy of pDC identification. Here, we thoroughly investigated BDCA-4 expression dynamics on pDCs and other PBMC subsets following prolonged activation with Toll-like receptor (TLR) 7 and TLR9 agonists. Our flow cytometry analysis revealed a significant increase in BDCA-4-positive non-pDC populations after extended stimulation, primarily corresponding to CD14^+^ monocytes. To overcome this limitation, we performed a gating strategy combining BDCA-4 positivity with a cocktail of non-pDC markers, enabling the exclusion of non-pDCs and accurate identification of pDCs. This approach enables the reliable identification of pDCs within heterogeneous cell populations using only two fluorescent channels in healthy conditions and even during strong activation or pathological states characterized by chronic inflammation.

## 1. Introduction

Plasmacytoid dendritic cells (pDCs) represent a unique subset of dendritic cells (DCs) specialized in the rapid and robust production of type I interferons (IFNs) [1,2]. Owing to their exceptional capacity to secrete vast quantities of type I IFNs, pDCs play a pivotal role in the pathogenesis of numerous human diseases [3,4]. The large amounts and diverse subtypes of type I IFNs they produce exert potent antiviral effects and have also been shown to display antitumor activity. However, excessive pDC activation and uncontrolled type I IFN overproduction can lead to tissue damage and the initiation of autoimmune responses. Consequently, pDCs contribute to the pathogenesis of various autoimmune disorders, including systemic lupus erythematosus (SLE) [5], psoriasis [6], Sjögren’s syndrome [7], and other autoimmune conditions [8]. Thus, the investigation of pDC immunological functions is of particular importance within the broader field of DC research.

The number of pDCs in peripheral blood may hold prognostic value in several disease contexts. Among the different DC subsets, pDCs have proven to be the most reliable indicators of short-term mortality in critically ill patients. A reduced pDC count was identified as a strong and independent prognostic factor for 30-day mortality, and low levels were also associated with elevated inflammatory markers, septic shock, respiratory failure, and increased severity of organ dysfunction [9]. In patients with COVID-19, circulating pDC numbers were likewise significantly decreased in more severe cases, and lower levels correlated with poor outcomes. Conversely, recovery of pDC counts paralleled clinical improvement, suggesting that pDC dynamics may serve as a prognostic factor for disease course [10]. In the context of solid tumors, such as colorectal cancer [11] and breast cancer [12], higher levels of tumor-infiltrating pDCs have been associated with improved survival rates in multiple studies. In breast cancer, elevated circulating pDC counts have also been identified as a positive prognostic marker [13]. In autoimmune diseases such as SLE and psoriasis, peripheral blood pDC numbers are often reduced due to their migration into inflamed tissues, where they actively contribute to disease pathogenesis. Thus, pDC activity and migratory patterns may also be linked to disease progression in these conditions [14,15].

The aforementioned data highlight that the circulating pDC count alone can provide valuable insights into disease activity, progression, and prognosis across various conditions. However, one of the challenges in pDC research lies in the identification of circulating pDC populations, as the expression of their specific surface markers, such as Blood Dendritic Cell Antigen (BDCA)-2 and BDCA-4, is dynamically changed depending on the maturation and activation status of the cells. BDCA-2 expression is completely downregulated upon pDC maturation or activation [16]. Moreover, BDCA-2 undergoes rapid internalization following labeling with monoclonal antibodies, and its ligation affects pDC functional activity by inhibiting IFN-α production and, consequently, TLR-mediated activation [17,18]. Recent single-cell RNA sequencing data further indicate that BDCA-2 is also expressed on DC progenitors and rare DC subsets, such as DC5/Axl^+^Siglec6^+^ DC (AS-DC) populations [19,20]. In contrast, the use of BDCA-4 for pDC identification offers the advantage that its expression does not decrease upon maturation—on the contrary, it increases—ensuring that the pDC-identifying signal is retained [16]. Our group has previously demonstrated that BDCA-4 positivity, combined with light scatter parameters, can reliably identify pDCs in whole peripheral blood without the need for additional surface markers [21]. Furthermore, when single-cell RNA sequencing analyses identified BDCA-4, similarly to BDCA-2, on circulating DC progenitors and other rare DC subpopulations [19,20], we showed that secondary gating based on light scatter parameters following BDCA-4 selection can effectively exclude pre-DC populations as well [22]. Nevertheless, identification of pDCs from peripheral blood mononuclear cells (PBMCs) using this approach is most reliable when targeting resting pDCs or cells from healthy donors after short-term activation. Caution is warranted for prolonged activation or under inflammatory conditions, as BDCA-4 expression patterns may change within heterogeneous cell populations.

The aim of the present study was to comprehensively investigate the changes in BDCA-4 expression on pDCs within PBMCs in response to various activation stimuli and pathological conditions, and to determine which gating strategies allow reliable identification of a pure pDC population based on BDCA-4. Our results demonstrate that following prolonged activation with different Toll-like receptor (TLR) ligands, the proportion of BDCA-4-positive cells markedly increases within PBMCs, suggesting that BDCA-4 can be induced on other cell types, thereby compromising the reliability of BDCA-4-based pDC identification. Under such conditions, accurate pDC identification requires the combined assessment of multiple parameters, incorporating markers that are not exclusively expressed on pDCs alongside BDCA-4, which allows the exclusion of non-pDC populations that may express BDCA-4. The pDC identification strategy we propose enables the isolation of a pure pDC population using only two fluorescent channels, both in highly activated heterogeneous cell populations from healthy individuals and in PBMC samples from patients with chronic inflammatory conditions. This approach prevents false data arising from contamination by non-pDCs, thereby ensuring accurate assessment of pDC numbers and functional activity across various physiological and pathological states.

## 2. Results

### 2.1. Altered BDCA-4 Expression in PBMCs Following Prolonged TLR7/9 Activation

**Figure 1:**
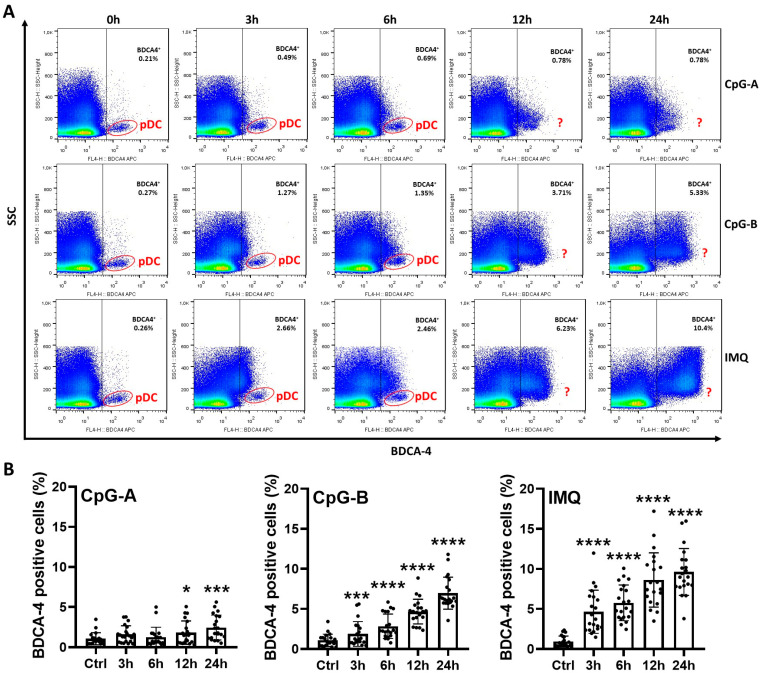
Activation-induced BDCA-4 expression on non-pDC cell populations limits BDCA-4-based pDC identification in PBMCs. PBMCs were separated from peripheral blood of healthy donors, then the cells were activated with 1 μM CpG-A or 1 μM CpG-B or 5 μg/mL IMQ for 3, 6, 12 or 24 h and total BDCA-4 positivity was determined by flow cytometry (**A**,**B**). Representative dot plots are shown in (**A**). Square gates indicate BDCA-4^+^ cells, and the red oval gates indicate the pDC population within the BDCA-4^+^ cells. Red question marks represent situations where the pDC population cannot be distinguished (**A**). Data are represented as means ± SD of 21 individual experiments (**B**) and analyzed using one-way ANOVA followed by Bonferroni’s post hoc test. * *p* < 0.05 *** *p* < 0.001; **** *p* < 0.0001 vs. control (ctrl). IMQ: imiquimod, SSC: side scatter.

In our laboratory experiments, peripheral blood samples were first collected from healthy donors, and mononuclear cells were subsequently isolated using Ficoll-Paque Plus density gradient centrifugation. The cells were then activated with various synthetic Toll-like receptor (TLR) agonists. Since pDCs selectively express TLR7 and TLR9 in their endosomes, cells were stimulated with two different TLR9 agonists, CpG-A and CpG-B, as well as the TLR7 agonist Imiquimod, for 3, 6, 12, and 24 h. CpG-A and CpG-B are unmethylated oligonucleotide sequences that, although acting through the same receptor, differ in their intracellular targeting: CpG-A primarily binds TLR9 in early endosomes and predominantly induces type I IFN secretion, whereas CpG-B localizes mainly to late endosomes, serving as a stronger inducer of nuclear factor kappa B (NF-κB) activation, pro-inflammatory cytokine production (interleukin-[IL]-6, tumor necrosis factor [TNF]-α), and cell maturation [23]. Imiquimod is a synthetic single-stranded RNA that mimics viral nucleic acids and strongly enhances both type I IFN and pro-inflammatory cytokine production in addition to inducing cell maturation [24]. Activated cells were stained with BDCA-4-specific antibodies and analyzed by flow cytometry to assess the feasibility of BDCA-4-based pDC identification using different TLR agonists in a time-dependent manner (Figure 1A). The pDC population could be clearly delineated based on side scatter (SSC) parameters and BDCA-4 positivity in untreated samples, as well as in samples treated for 3 and 6 h with CpG-A, CpG-B, or Imiquimod. However, for all three activators, the proportion of BDCA-4-positive cells in PBMCs markedly increased after 12 and 24 h of incubation. Therefore, the previously well-defined pDC population could no longer be reliably identified solely based on SSC and BDCA-4 positivity (Figure 1A).

Following 12 and 24 h of CpG-A stimulation, the proportion of BDCA-4-positive cells increased more than threefold compared to untreated samples, although still remaining below 1%. In PBMCs activated with CpG-B for 12 and 24 h, BDCA-4-positive cells accounted for approximately 3–5%, while in Imiquimod-treated samples, the proportion reached approximately 6–10% (Figure 1A,B). Given that pDCs normally comprise less than 1% of total PBMCs [25], these results suggest that the higher proportion of BDCA-4 positivity is likely due to induction of BDCA-4 on other cell types following activation. Overall, these findings indicate that in PBMCs, pDC identification based solely on BDCA-4 positivity is not feasible after more than 12 h of stimulation with pDC-specific endosomal TLR agonists.

### 2.2. BDCA-4-Based pDC Identification Complemented with Non-pDC Markers Yields a Well-Defined pDC Population Even After Prolonged TLR7/9 Activation

pDC identification in PBMCs based solely on BDCA-4 positivity can be advantageous, as it requires only one pDC-specific antibody and a single fluorescent channel [21]. However, as demonstrated by our previous results, this approach is primarily suitable for unstimulated samples or short-term cell activation, since prolonged TLR stimulation induces changes in BDCA-4 expression within the PBMC population. As a next step, we aimed to identify pDCs using a BDCA-4 marker in combination with an antibody cocktail designed to exclude non-pDC populations. This cocktail contains antibodies against surface markers that are negative on pDCs, including lineage (LIN) markers (CD3, CD14, CD16, CD19, CD20, and CD56) as well as CD2, CD5, CD33, and AXL, which help eliminate contaminating pre-DCs and other DC subpopulations. This strategy also considers recent single-cell RNA sequencing data showing that BDCA-4 is expressed on DC progenitors and rare DC subsets, such as the DC5/AS-DC population [19,20].

**Figure 2:**
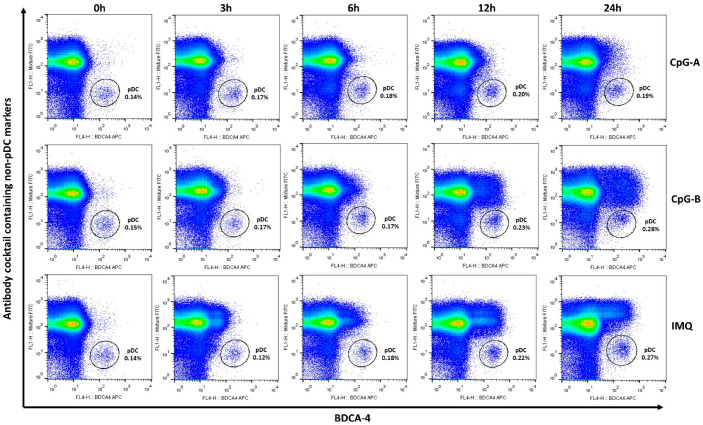
Using BDCA-4 in combination with an antibody cocktail containing non-pDC markers enabled clear separation of the pDC population from PBMCs, even after prolonged activation. PBMCs were separated from peripheral blood of healthy donors, then the cells were activated with 1 μM CpG-A or 1 μM CpG-B or 5 μg/mL IMQ for 3, 6, 12 or 24 h. After activation cells were stained for BDCA-4 and an antibody cocktail containing non-pDC markers and pDC population was determined by flow cytometric analysis. Representative dot plots are shown from 21 independent experiments. Gates indicate the pDC population which is positive for BDCA-4 but negative for non-pDC markers of the antibody cocktail. IMQ: imiquimod.

Accordingly, pDC identification in PBMCs was performed using one fluorescent channel to determine BDCA-4 positivity and a second channel to exclude non-pDC populations using the antibody cocktail (Figure 2). Using this approach, we were able to reliably identify a clearly delineated pDC population even after prolonged TLR activation for all three stimuli (Figure 2).

Following gating with this method, backgating onto previously described forward and side scatter (FSC–SSC) parameters was performed on the gated pDC population [21,22]. A detailed description of the gating strategy for the pDC population is provided in the Supplementary Materials (Supplementary Figure S1). In the gated pDC population, both the percentage of pDCs and the median fluorescence intensity (MFI) of BDCA-4 were determined in untreated and activated cells. (Supplementary Figure S2A–F). In CpG-A–treated samples, only 24 h activation caused a significant increase in the proportion of BDCA-4-positive pDCs (Supplementary Figure S2A), whereas in CpG-B– and Imiquimod-activated cells, the percentage of BDCA-4-positive pDCs was significantly elevated at 6, 12, and 24 h, remaining within the typical pDC proportion range in PBMCs (Supplementary Figure S2B,C). Analysis of BDCA-4 fluorescence intensity in the gated pDC population revealed a significant decrease in BDCA-4 expression on pDCs following CpG-A activation, which became more pronounced with longer incubation times, although complete downregulation was not observed even after 24 h (Supplementary Figure S2D). In contrast, CpG-B–activated pDCs showed increased BDCA-4 surface expression at 6, 12, and 24 h (Supplementary Figure S2E). Similar results were observed in Imiquimod-activated pDCs, with significant increases in BDCA-4 levels at 6 and 12 h; however, after 24 h of Imiquimod stimulation, BDCA-4 levels were no longer significantly elevated compared to untreated samples (Supplementary Figure S2F).

These results indicate that BDCA-4 expression on pDCs is dynamically changing upon activation. Ligands primarily inducing type I IFN may decrease BDCA-4 expression, whereas TLR agonists driving pDC maturation tend to enhance BDCA-4 surface levels. Importantly, unlike BDCA-2, which is strongly downregulated upon pDC maturation, BDCA-4 maintains high expression even after prolonged activation, making it a more reliable marker for pDC identification.

### 2.3. Prolonged TLR Activations Induce BDCA-4 Expression in Non-pDC Populations of PBMC Cultures

**Figure 3:**
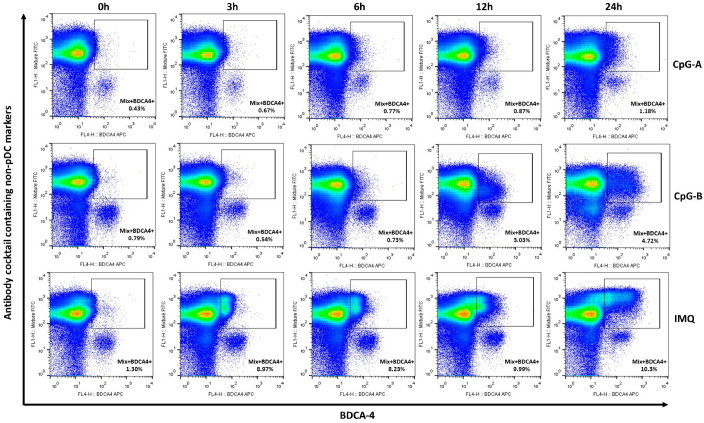
Non-pDC markers combined with BDCA-4 revealed the induction of BDCA-4+ non-pDC population after prolonged TLR activation in PBMCs. PBMCs were separated from peripheral blood of healthy donors, then the cells were activated with 1 μM CpG-A or 1 μM CpG-B or 5 μg/mL IMQ for 3, 6, 12 or 24 h. After activation, cells were stained for BDCA-4 and with an antibody cocktail containing non-pDC markers, and the BDCA-4-positive non-pDC population was determined by flow cytometry. Representative dot plots are shown from 21 independent experiments. Gates indicate the BDCA-4-positive non-pDC population, which is also positive for the non-pDC markers included in the antibody cocktail. IMQ: imiquimod.

As previously shown, prolonged activation of PBMC cultures with endosomal TLR ligands markedly increases the proportion of total BDCA-4-positive cells in PBMCs, thereby complicating pDC identification based solely on BDCA-4 (Figure 1A,B). However, when pDC identification in PBMCs is performed using BDCA-4 positivity in combination with an antibody cocktail designed to exclude non-pDC populations, as demonstrated above, the pDC population can be clearly distinguished from a larger BDCA-4-positive population that also displays positivity for the non-pDC markers in the antibody cocktail (Figure 3).

The proportion of BDCA-4-positive non-pDC cells in PBMCs increased following activation with all three TLR agonists (Supplementary Figure S3A–C). The smallest effect was observed with CpG-A, which only induced a significant increase in this population after 24 h compared to untreated cells (Supplementary Figure S3A). In contrast, both CpG-B and Imiquimod induced a significantly higher proportion of BDCA-4-positive non-pDC cells after just 3 h gradually increased over time (Supplementary Figure S3B,C). Analysis of BDCA-4 fluorescence intensity in this population revealed that 12- and 24 h treatments with CpG-B and Imiquimod produced substantial increases in BDCA-4 expression, most pronounced in the 24 h Imiquimod-treated samples (Supplementary Figure S3D–F).

These results indicate that the endosomal TLR agonists used to activate pDCs can also induce BDCA-4 expression on other cell types within PBMCs, particularly after prolonged activation, which complicates BDCA-4-based pDC identification in PBMC cultures.

### 2.4. BDCA-4 Upregulation in PBMCs May Be Associated with CD14^+^ Monocytes Following TLR7/9 Activation

**Figure 4:**
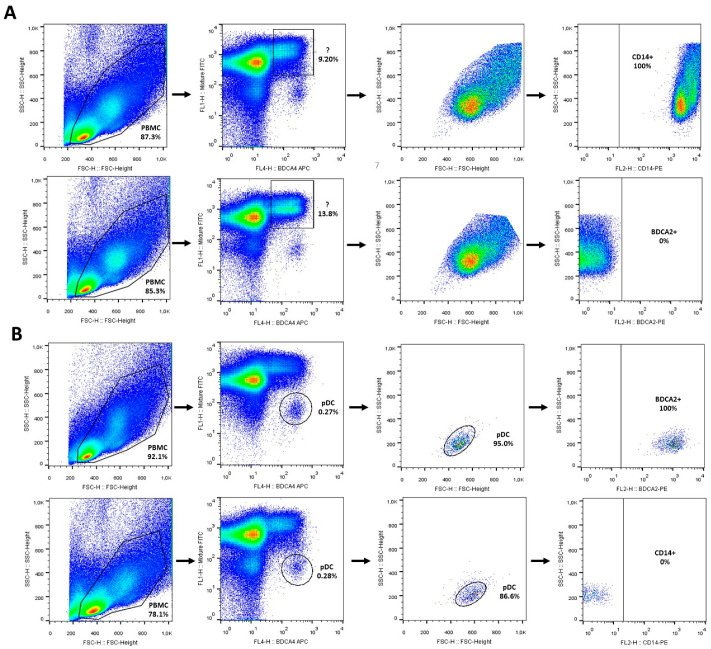
Characterization of BDCA-4 positive non-pDC and pDC populations. PBMCs were isolated from the peripheral blood of healthy donors and activated with 5 μg/mL IMQ for 24 h. After activation, cells were stained for BDCA-4 and with an antibody cocktail containing non-pDC markers (**A**,**B**) to distinguish the BDCA-4 positive pDC population from the BDCA-4 positive non-pDC population using flow cytometry. The non-pDC population was defined as cells positive for both BDCA-4 and the non-pDC antibody cocktail (**A**), whereas pDCs were defined as BDCA-4 positive but negative for non-pDC markers (**B**). Subsequently, the distribution of cells within the gated populations was analyzed by backgating on light scatter parameters or on CD14 and BDCA-2 positivity. Representative dot plots are shown in (**A**,**B**). IMQ: imiquimod.

Next, we investigated which cell types in PBMCs upregulate BDCA-4 following prolonged endosomal TLR7 and TLR9 activation. We analyzed the gated population that was positive for both BDCA-4 and the non-pDC antibody cocktail. Based on FSC–SSC parameters, this population fell within the PBMC monocyte gate, allowing us to exclude lymphocyte origin based on light scatter characteristics (Figure 4A). CD14 is one of the most classical markers of monocytes; analysis of this population revealed 100% CD14 positivity (Figure 4A). To assess potential pDC contamination, we examined BDCA-2, another pDC-specific marker, and found the cells were entirely negative (Figure 4A). Subsequently, we verified that the BDCA-4-positive and non-pDC marker-negative gated population indeed corresponded to pDCs by analyzing BDCA-2 expression, which was 100% positive, while CD14 expression was completely negative (Figure 4B).

These findings indicate that following endosomal TLR7 and TLR9 activation, BDCA-4 induction primarily occurs on CD14^+^ cells in PBMC cultures, which, based on FSC–SSC light scatter parameters, most likely correspond to classical, intermediate, or non-classical monocytes.

### 2.5. Evaluation of BDCA-4-Based Identification of pDCs in Psoriasis Patients

Next, we sought to investigate whether BDCA-4-based pDC identification could be applied under pathological conditions and how BDCA-4 induction on other cell populations within PBMCs affects the accuracy of BDCA-4-based pDC detection. To this end, PBMCs were isolated from the peripheral blood of patients with plaque-type psoriasis, a chronic autoimmune disease characterized by persistent inflammation, elevated type I IFN production, and pDC overactivation [6]. In addition, circulating pDC numbers in psoriasis patients are generally reduced due to their migration into inflamed skin lesions [6].

**Figure 5:**
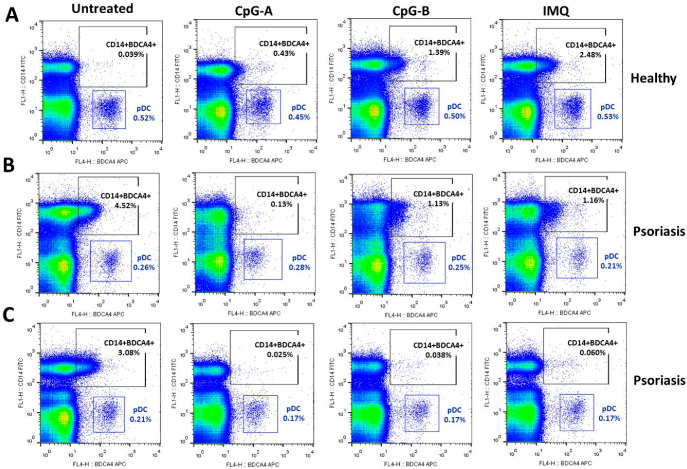
Investigation of the distribution of BDCA-4 positive pDC and non-pDC populations in PBMCs from psoriasis patients. Peripheral blood samples were collected from healthy volunteers (**A**) and psoriasis patients (**B**,**C**). PBMCs were isolated using Ficoll-Paque gradient centrifugation and then activated with 1 μM CpG-A, 1 μM CpG-B, or 5 μg/mL IMQ for 3 h. After activation, cells were stained for BDCA-4 and CD14 to distinguish the BDCA-4 positive pDC population from the BDCA-4 positive non-pDC population by flow cytometry. The non-pDC population was defined as cells positive for both BDCA-4 and CD14, whereas pDCs were defined as BDCA-4 positive but CD14 negative. Representative dot plots are shown from 14 independent experiments. IMQ: imiquimod.

Since we previously observed that BDCA-4 is primarily induced on CD14^+^ cells following activation, cells were stained for both BDCA-4 and CD14, and the BDCA-4-positive, CD14-negative population was defined as pDCs. Using these two surface markers, the pDC population was clearly identifiable in both healthy donor controls and psoriasis patient samples (Figure 5A–C). In healthy donors, BDCA-4 positivity on CD14^+^ cells was minimal in untreated samples, whereas, as previously shown, TLR activation increased the proportion of BDCA-4^+^CD14^+^ double-positive cells (Figure 5A). Interestingly, in psoriasis patients, BDCA-4^+^CD14^+^ cells were present at a higher proportion even in unstimulated samples (Figure 5B,C). Upon TLR agonist stimulation, a 3 h activation significantly reduced the proportion of BDCA-4^+^CD14^+^ cells (Figure 5B) or nearly completely suppressed BDCA-4 expression on CD14^+^ cells (Figure 5C).

The BDCA-4^+^CD14^−^ population defined as pDCs showed a reduced percentage compared to healthy donors in both unstimulated and activated samples (Supplementary Figure S4A). However, BDCA-4 fluorescence intensity on pDCs did not differ between healthy and psoriasis samples under either unstimulated or activated conditions. CpG-A slightly decreased, while CpG-B and Imiquimod significantly increased BDCA-4 expression on pDCs, independent of donor disease status (Supplementary Figure S4B). Statistical analysis of the BDCA-4^+^CD14^+^ population revealed significant differences between healthy and patient samples only in control conditions. In unstimulated samples, psoriasis patients displayed a higher proportion of BDCA-4^+^CD14^+^ cells compared to healthy donors. Upon activation, this population increased significantly in healthy donors but decreased in psoriasis patients relative to baseline. This opposing pattern likely explains why no significant differences were observed between healthy and patient groups for BDCA-4^+^CD14^+^ percentages under TLR stimulation (Supplementary Figure S4C). Analysis of BDCA-4 surface expression within the BDCA-4^+^CD14^+^ population showed consistently lower fluorescence intensity in psoriasis patients compared to healthy donors in both unstimulated and activated samples (Supplementary Figure S4D).

These results suggest that in pathological conditions, BDCA-4, a pDC-specific surface molecule, may appear on CD14^+^ cells even without prior in vitro activation. Therefore, BDCA-4-based pDC identification should always be combined with additional non-pDC markers under such conditions. Overall, our findings indicate that, with exclusion of the CD14^+^ population, BDCA-4-based pDC identification remains a reliable method for detecting pDCs in PBMC samples from both healthy individuals and patients with chronic inflammation.

These results are in line with findings from a conference abstract published in *Annals of the Rheumatic Diseases* [26], which reported that BDCA-2, BDCA-4, and CD123 markers are not exclusively pDC-specific and may also appear on CD14^+^ cells after 24 h in vitro culture in both healthy and SLE patients. Additionally, purified CD14^+^ cells differentiated into M1 and M2 macrophages retained surface pDC markers [26]. In the present study, we did not have the opportunity to examine samples from patients with SLE. However, to further explore BDCA-4 (gene symbol: *NRP1* [*Neuropilin-1*]) and BDCA-2 (gene symbol: *CLEC4C* [*C-type Lectin Domain Family 4 Member C*]) expression in SLE, we analyzed raw RNA sequencing counts from a published study [27] available in the Gene Expression Omnibus (Supplementary Figure S5A,B). Differential gene expression analyses were performed for pDCs, CD14^+^ monocytes, and conventional DCs (cDCs) in patients with and without an IFN signature. No significant differences were observed in *NRP1* (Supplementary Figure S5A) or *CLEC4C* (Supplementary Figure S5B) expression between healthy controls and SLE patients with different disease activity. While pDCs exhibited the highest expression levels for both genes among cell types, *NRP1* expression in monocytes approached levels seen in pDCs, whereas *CLEC4C* remained low in monocytes relative to pDCs. In our flow cytometry analysis, BDCA-2 positivity was not detected in the BDCA-4^+^CD14^+^ population. However, gene expression does not always reflect protein abundance; thus, BDCA marker surface expression should be confirmed at the protein level in SLE and other pathological conditions. Considering disease subtype, activity, and progression, BDCA marker expression is likely dynamic, emphasizing the need for integrated gene- and protein-level analyses correlated with clinical parameters in future studies.

## 3. Discussion

pDCs exert profound influence on both physiological and pathological processes through their exceptional ability to produce type I IFNs. Nevertheless, working with pDCs presents several challenges. First, pDCs are present at very low frequencies in peripheral blood and are short-lived in vitro due to rapid apoptosis [28]. Second, their hybrid lymphoid and myeloid characteristics [29] require careful identification within heterogeneous cell populations.

Early studies identified two human pDC-specific markers, BDCA-2 and BDCA-4, suitable for detecting this population in various body compartments [16,30]. BDCA-2, previously considered a specific marker for pDCs, is expressed exclusively on these cells and has been widely used to identify them in blood circulation and various tissues. In contrast, BDCA-4 is primarily a peripheral blood-specific marker, as it is also expressed on other cells in tissues, including neurons, endothelial cells, developing thymocytes, thymic epithelial cells (TECs), and other DCs [31]. Although BDCA-2 appears to be a more reliable pDC-specific marker, its expression is strongly downregulated during pDC maturation or activation [16]. BDCA-2 is a C-type lectin receptor that recognizes carbohydrate patterns on viruses, bacteria, and fungi. Lacking an intrinsic signaling domain, it signals via the FcεRIγ adapter, activating the Spleen tyrosine kinase (Syk)—B-cell linker protein (BLNK)—Phospholipase C gamma 2 (PLCγ2) cascade, which inhibits type I IFN production by pDCs and modulates immune responses [32]. Consequently, BDCA-2 ligation negatively regulates TLR-mediated pDC activation, including the production of IFN-α, IFN-β, and IL-6, as well as the capacity of pDCs to induce T cell proliferation [32]. Furthermore, anti-BDCA-2 antibodies have been explored as therapeutic agents in autoimmune diseases, as they inhibit IFN production by pDCs [18]. Collectively, these findings indicate that BDCA-2-based identification of mature or activated pDCs may be challenging due to reduced surface expression. Moreover, using BDCA-2 antibodies for cell isolation or functional assays may alter pDC activity, including diminished type I IFN production. In contrast to BDCA-2, BDCA-4 expression increases with pDC maturation [16], and BDCA-4 ligation exerts a considerably weaker inhibitory effect, inducing only partial suppression of IFN-α production by pDCs [33]. BDCA-4 also functions as a surface co-receptor involved in cell–matrix and cell–cell interactions, as well as in the regulation of cell motility and migration under both physiological and pathological conditions [34,35]. It is therefore plausible that BDCA-4 contributes to directing pDC migration to lymphoid organs or sites of inflammation, a process critical for immune responses to infections or other inflammatory stimuli. This may explain the observed upregulation of BDCA-4 during pDC maturation or activation, as it is required for subsequent migratory events, in contrast to BDCA-2, which primarily regulates pDC IFN production, and is downregulated upon activation to allow proper IFN responses.

Our group previously demonstrated that in whole blood—where only red blood cells are removed prior to analysis—pDCs can be reliably identified using BDCA-4 and backgating on FSC-SSC parameters in both untreated and 24 h Imiquimod-stimulated samples [21]. However, in the present study, we show that in PBMCs—where mononuclear cells are isolated via Ficoll-Paque Plus gradient centrifugation, excluding granulocytes—prolonged TLR ligand stimulation for 12–24 h prevents clear pDC identification based solely on BDCA-4. While BDCA-4 positivity is largely restricted to pDCs in untreated and 3–6 h activated samples, longer incubation results in a marked increase in BDCA-4-positive cells within the BDCA-4 population, suggesting that additional PBMC subsets may upregulate BDCA-4 upon activation. By 2017, single-cell RNA sequencing revealed that both BDCA-2 and BDCA-4 are also expressed on other cell types, notably DC precursors and rare DC subpopulations such as DC5/AS-DCs [19,20]. Accordingly, negative pDC markers, including CD2 [36], CD5 [37], AXL [38], and CD33 [39], are now recommended to assess the composition and purity of pDC populations [40]. Using this approach, we assembled an antibody cocktail comprising lineage markers and the aforementioned pre-DC markers; combined with BDCA-4 staining, this strategy enabled reliable pDC identification even after prolonged TLR stimulation. Within this gated pDC population, we observed that the number of BDCA-4-positive cells increases primarily after 12–24 h of TLR activation, which may reflect limited activation-induced proliferation of pDCs. Importantly, BDCA-4 expression levels vary depending on the TLR activation signal. CpG-A, which induces strong type I IFN production, significantly decreases BDCA-4 expression on pDCs over time. In contrast, TLR ligands that induce lower type I IFN production but promote pDC maturation, such as CpG-B and Imiquimod, significantly increase BDCA-4 expression. These findings are consistent with previous observations that BDCA-4 expression is enhanced in the presence of IL-3, which also supports pDC maturation [16]. Notably, not all activation signals consistently upregulate surface BDCA-4 on pDCs; stimuli that induce IFN-producing pDCs without full maturation may decrease BDCA-4 expression, similarly to BDCA-2 downregulation, potentially facilitating efficient IFN production by limiting interference from BDCA-associated pathways [17]. Importantly, although BDCA-4 expression decreases following CpG-A stimulation, it is not entirely lost from the cell surface, unlike BDCA-2 [16], and can therefore still be employed for pDC identification.

In our study, we further observed that prolonged TLR7 and TLR9 stimulation in PBMCs induces BDCA-4 expression primarily on CD14^+^ cells, with the most pronounced upregulation occurring following CpG-B and Imiquimod activation. Within the PBMC fraction, CD14 expression is largely restricted to the monocyte population, encompassing classical, intermediate, and non-classical subsets that exhibit varying expression intensities [41]. CD14 can also be expressed on monocyte-like myeloid-derived suppressor cells (M-MDSCs), although these cells are predominantly present under pathological conditions [42]. In our experiments, the proportion of BDCA-4^+^CD14^+^ cells following TLR stimulation was approximately 5–10%, roughly corresponding to the average monocyte fraction in PBMCs (~10%) [43], and their FSC-SSC light scatter profiles were consistent with a monocytic phenotype. Monocytes express TLR7 and TLR9 receptors [44], suggesting that the TLR ligands we applied could directly activate monocytes in addition to pDCs, likely contributing to the induction of BDCA-4 expression. BDCA-4 upregulation on monocytes may activate an adaptive virus-sensing mechanism in response to endosomal TLR agonists mimicking viral nucleic acids, as BDCA-4 has been implicated in antiviral responses and in viral recognition and internalization [45,46]. As mentioned above, BDCA-4 ligation on pDCs has been reported to suppress TLR-mediated IFN-α production [32]. While direct evidence for a similar mechanism in monocytes or macrophages is currently lacking, it is noteworthy that monocytes are also capable of producing type I IFNs in response to viral stimuli [47]. This raises the possibility that BDCA-4 upregulation upon activation could influence their antiviral or immunomodulatory responses, which warrants future investigation. Moreover, BDCA-4 has several known ligands [48] that facilitate cell–cell interactions. Such interactions may contribute to communication and migration of both pDCs and activated monocytes into inflamed tissues, representing a potential area of functional overlap. Although BDCA-4 expression has not been previously reported on resting monocytes [49], its expression on macrophages, which represent the tissue-resident counterparts of monocytes, is well documented. BDCA-4 expression on macrophages has been associated with immunoregulatory and pro-angiogenic functions, often linked to M2-like polarization within the tumor microenvironment [50,51]. Furthermore, BDCA-4 expressed by adipose tissue macrophages has been shown to regulate obesity-associated inflammation, where BDCA-4 positive macrophages dampen inflammatory responses and protect against metabolic dysfunction [52]. Thus, it is conceivable that BDCA-4 engagement could also contribute to the modulation of inflammatory responses in monocytes/macrophages, albeit via distinct signaling pathways from those operating in pDCs. However, the precise functional role of BDCA-4 on activated monocytes remains to be elucidated.

Taken together, our results indicate that, upon activation, CD14^+^ cells—presumably monocytes—also upregulate BDCA-4 expression. Therefore, when identifying pDCs, attention should be given not only to CD33^+^ pre-DCs and AXL^+^ DC5/AS-DCs but also to the CD14^+^ population. Our antibody cocktail included lineage markers, including anti-CD14, which likely facilitated the discrimination of pDCs in prolonged TLR-activated samples.

Previously, in whole blood containing granulocytes, we did not observe the emergence of BDCA-4^+^CD14^+^ cells that could interfere with BDCA-4-based pDC gating even after 24 h Imiquimod stimulation [21]. Several studies indicate that granulocyte-derived factors can inhibit monocyte activation, maturation, and proinflammatory cytokine production [53,54,55,56]. This suggests that monocyte activation, maturation, and consequent BDCA-4 induction may be suppressed in heterogeneous populations containing granulocytes, although further studies are needed to confirm this hypothesis.

We next investigated how BDCA-4 positivity is altered under pathological conditions. In PBMCs from psoriasis patients, we analyzed both BDCA-4^+^CD14^+^ cells and BDCA-4^+^CD14^−^ cells, the latter defined as pDCs. The role of pDCs in psoriasis pathogenesis is well-documented, with multiple studies demonstrating their hyperactivation and critical involvement in early disease phases [6,14]. Consistent with prior reports, circulating pDCs are generally reduced in psoriasis patients due to migration into inflamed skin lesions [57]. Accordingly, we observed a lower percentage of BDCA-4^+^ pDCs in psoriasis patients compared to healthy controls. Notably, BDCA-4^+^CD14^+^ cells were detectable in untreated patient samples, whereas in healthy donors these cells appeared primarily upon in vitro activation. Interestingly, in vitro stimulation of psoriasis PBMCs markedly reduced—or even completely eliminated—the BDCA-4^+^CD14^+^ population. Moreover, BDCA-4 expression on this population was significantly lower in both untreated and activated patient samples relative to healthy controls. This may reflect in vivo pre-activation or an exhausted state of BDCA-4^+^CD14^+^ cells in psoriasis, leading to decreased BDCA-4 expression upon subsequent TLR7/9 stimulation, potentially through negative feedback, receptor internalization, or transient differentiation/redistribution into other subtypes. Chronic inflammatory environments may also induce epigenetic or transcriptional suppression at the BDCA-4 locus. Importantly, BDCA-4 expression remained stable on pDCs, and as demonstrated, combining BDCA-4 with CD14 staining facilitated the accurate discrimination of pDCs from non-pDCs even in psoriasis samples. It is important to note that a previous conference abstract [26] also reported that BDCA-2, BDCA-4, and CD123 are not strictly pDC-specific and can appear on CD14^+^ cells after 24 h in vitro culture in both healthy donors and SLE patients. In SLE samples, we observed at the mRNA level that BDCA-4 (*NRP1*) expression in monocytes was much closer to that in pDCs than BDCA-2 (*CLEC4C*). These findings indicate that BDCA-4, traditionally considered a pDC-specific marker, can also be upregulated on monocytes in pathological conditions.

Overall, our study highlights that BDCA-4 displays considerably more stable expression on pDCs compared to BDCA-2, which is rapidly downregulated during pDC maturation [16], or CD123, whose surface levels may also decrease after pDC activation [58] or under microenvironmental influences [59]. Nonetheless, BDCA-4 remains a reliable marker for pDC identification under prolonged activation or pathological conditions when combined with appropriate negative markers to exclude non-pDC populations. Moreover, the induction of BDCA-4 on CD14^+^ monocytes under pathological or long-term stimulatory conditions suggests a potential novel regulatory role of this molecule in monocyte activation and immune modulation, which warrants further functional investigation. Together, these findings not only enhance strategies for pDC detection but also highlight the potential role of BDCA-4 in monocyte biology and immune regulation.

## 4. Materials and Methods

### 4.1. Collection of Human Blood Samples and Isolation and Activation of PBMCs

Heparinized, leukocyte-enriched buffy coat samples from 21 healthy volunteers were collected in accordance with the Declaration of Helsinki and approved by the National Blood Transfusion Service and the Regional and Institutional Ethics Committee of the University of Debrecen, Faculty of Medicine (OVSzK 3572-2/2015/5200, Hungary). Peripheral blood mononuclear cells (PBMCs) from healthy donors were isolated by Ficoll-Paque Plus (Cytiva, Uppsala, Sweden, Cat. No. 17144003) density gradient centrifugation. Patient blood samples were provided to us by the Laboratory of Regenerative Medicine and Cellular Pharmacology (Department of Dermatology and Allergology, University of Szeged). The collection of peripheral blood samples complied with the guidelines of the Helsinki Declaration and was approved by the National Public Health and Medical Officer Service (NPHMOS) and the National Medical Research Council (administrative number: 13740-5/2021/EÜIG and 4969; 90/2021-SZTE IKEB, protocol code: PSO-CELL-01), which follows the EU Member States’ Directive 2004/23/EC and GDPR on presumed written consent practice for tissue collection. Men and women aged 25–65 years with plaque psoriasis and a Psoriasis Area and Severity Index (PASI) > 15 were included. Patients receiving systemic therapies (biological or traditional) or total body phototherapy were excluded. In total, blood samples from 14 patients with psoriasis and 14 age- and sex-matched healthy donors (40–55 years) were processed for analysis. The healthy donors served as controls. Peripheral blood (25 mL) was collected into 10 mL BD Vacutainer^®^ tubes containing spray-coated K2EDTA (BD Biosciences, Milpitas, CA, USA, Cat. No. 366643), diluted 1:1 with physiological saline (0.9% NaCl, B. Braun, Melsungen, Germany), and PBMCs were isolated by Ficoll-Paque Plus gradient centrifugation. Freshly isolated PBMCs were seeded at 1 × 10^6^ cells per 500 μL RPMI 1640 medium (Sigma-Aldrich, St. Louis, MO, USA) supplemented with 10% heat-inactivated Fetal Bovine Serum (FBS; Life Technologies Corporation, Carlsbad, CA, USA), 2 mM L-glutamine, 100 U/mL penicillin, and 100 μg/mL streptomycin (all from Biosera, Nuaille, France). Cells were then stimulated with endosomal Toll-like receptor (TLR) agonists: 1 μM CpG-A (ODN 2216; Hycult Biotech, Uden, The Netherlands, Cat. No. HC4037), 1 μM CpG-B (ODN 2006; Hycult Biotech, Cat. No. HC4039), or 5 μg/mL Imiquimod (IMQ; InvivoGen, Toulouse, France, Cat. No. tlrl-imq) for 3, 6, 12, or 24 h. The selected concentrations and incubation times were based on our previous experimental data [22], which identified these conditions as optimal for endosomal TLR stimulation in pDCs within PBMC cultures.

### 4.2. Flow Cytometric Analysis

Untreated or activated PBMCs were centrifuged at 5000× *g* for 1 min, and supernatants were removed. Cells (1 × 10^6^) were resuspended in 20 μL FACS buffer, gently vortexed, and incubated with the following fluorochrome-conjugated antibodies: anti-BDCA-4-APC (CD304/Neuropilin-1; BioLegend, San Diego, CA, USA, Cat. No. 354506), anti-CD14-PE (BioLegend, Cat. No. 982508), anti-CD14-FITC (BioLegend, Cat. No. 301804), and anti-BDCA2-PE (CD303; BioLegend, Cat. No. 354204). For pDC identification, an antibody cocktail targeting lineage markers and pre-DC markers was used: LIN-FITC (BD Biosciences, Cat. No. 340546), CD2-FITC (Immunotech-Beckman Coulter, Marseille, France, Cat. No. IM0442), CD5-FITC (PharMingen-BD, San Diego, CA, USA, Cat. No. 555353), AXL-Alexa Fluor 488 (R&D Systems, Minneapolis, MN, USA, Cat. No. FAB154G), and CD33-FITC (eBioscience, San Diego, CA, USA, Cat. No. 11-0337-42). Each antibody was used at 0.5 μL per sample. After gentle vortexing, cells were incubated for 30 min at 4 °C in the dark. Cells were washed with FACS buffer and fixed in 2% paraformaldehyde (Alfa Aesar, Ward Hill, MA, USA, Cat. No. J61899). For determining cell viability, cells were stained with 7-aminoactinomycin D (7-AAD; working concentration: 1 µg/mL; Sigma-Aldrich, Cat. No. A9400) for 30 min at 4 °C in the dark prior to fixation. After incubation, cells were washed twice with FACS buffer to remove excess dye and then fixed.

Fluorescence intensities were measured on a FACSCalibur flow cytometer (BD Biosciences), and data were analyzed with FlowJo software v10 (TreeStar, Ashland, OR, USA). Approximately 600,000 events were acquired per sample, with a forward scatter (FSC) threshold of 160 to exclude debris. Live cells were pre-gated based on FSC and SSC parameters, followed by exclusion of doublets and 7-AAD-positive cells. pDCs were then identified as BDCA-4^+^ and cocktail-negative or CD14^−^ cells. Back-gating on light scatter parameters was performed to exclude non-pDCs, and cells in the FSC range of 400–600 were classified as pDCs as previously described [22]. The gating strategy used for pDC identification is shown in Supplementary Figure S1 of the Supplementary Materials.

### 4.3. Gene Expression Analysis

To assess *NRP1* and *CLEC4C* gene expression in SLE patient samples, raw RNA sequencing counts from the GSE149050 study [27] were downloaded from the Gene Expression Omnibus. Differential gene expression analyses were performed using EdgeR (Galaxy Version 3.36.0+galaxy5) on Galaxy Europe (https://usegalaxy.eu), with the following settings: -c ‘1.0’ -s ‘5’ -x -l ‘1.5’ -p ‘0.05’ -d ‘BH’ -n ‘TMM’ -b. Pairwise comparisons were conducted between healthy controls and SLE patients with (“IFN+”) or without (“IFN−”) an IFN response signature. The IFN response status (IFN+ vs. IFN−) was defined by hierarchical clustering of SLE patient classical monocyte bulk RNA-seq data, based on the top 50 most variable differentially expressed genes (DEGs) between SLE and healthy controls. Patients with high expression of interferon-stimulated genes (ISGs) and significant enrichment of a predefined IFN-signature gene set (IFN-20) were designated “IFN+”, while those with low ISG expression and clustering with the healthy controls were designated “IFN−”. The IFN-20 gene set consisted of *IFI27*, *IFI44*, *IFI44L*, *IFI6*, *IFIT1*, *IFIT2*, *IFIT3*, *IFITM1*, *IFITM3*, *ISG15*, *MX1*, *MX2*, *OAS1*, *OAS2*, *OAS3*, *OASL*, *RSAD2*, *SIGLEC1*, *USP18*, and *XAF1*, representing canonical ISGs previously associated with the SLE IFN response.

Cell types analyzed included pDCs, monocytes, and conventional DCs (cDCs). Normalized gene expression values generated by the program are shown in the graphs. The analysis included pDCs from 9 healthy donors, 11 SLE patients without an IFN signature, and 11 SLE patients with an IFN signature. For monocytes, samples from 23 healthy donors, 54 SLE patients without an IFN signature, and 39 SLE patients with an IFN signature were analyzed. In the case of cDCs, samples from 10 healthy donors, 11 SLE patients without an IFN signature, and 9 SLE patients with an IFN signature were included. Sample IDs are provided in Supplementary Material Table S1.

### 4.4. Statistical Analysis

Normality of data distributions was assessed using the Shapiro–Wilk test. Data are presented as mean ± SD and analyzed by one-way ANOVA followed by Bonferroni post hoc test. Analyses were performed using GraphPad Prism v6 (GraphPad Software, La Jolla, CA, USA). Differences were considered statistically significant at *p* < 0.05.

## Data Availability

The original contributions presented in this study are included in the article/Supplementary Materials. Further inquiries can be directed to the corresponding author.

## Supplementary Materials

The following supporting information can be downloaded at: https://www.mdpi.com/article/10.3390/ijms262210979/s1.

## Abbreviations

The following abbreviations are used in this manuscript:

APC	Antigen-Presenting Cell
AS-DC	Axl^+^Siglec6^+^ Dendritic Cell
BDCA	Blood Dendritic Cell Antigen
BLNK	B-cell linker protein
cDC	Conventional Dendritic Cell
CLEC4C	C-type Lectin Domain Family 4 Member C
DC	Dendritic Cell
FBS	Fetal Bovine Serum
FSC	Forward Scatter
IFN	Interferon
IL	Interleukin
IMQ	Imiquimod
LIN	Lineage cocktail
MFI	Median Fluorescence Intensity
M-MDSC	Monocytic Myeloid-Derived Suppressor Cell
NRP1	Neuropilin-1
PBMC	Peripheral Blood Mononuclear Cell
PASI	Psoriasis Area and Severity Index
PLCγ2	Phospholipase C gamma 2
pDC	Plasmacytoid Dendritic Cell
SLE	Systemic Lupus Erythematosus
SSC	Side Scatter
Syk	Spleen tyrosine kinase
TECs	Thymic Epithelial Cells
TLR	Toll-Like Receptor
TNF	Tumor necrosis factor
