# Supplementary material for: Flow Cytometric Challenges in Plasmacytoid Dendritic Cell (pDC) Identification: Limitation of BDCA-4 (CD304)-Based Gating
Source: Int J Mol Sci. 2025 Nov 13;26(22):10979. doi: 10.3390/ijms262210979 (PMC12652205; doi:10.3390/ijms262210979)
Supplement: Supplementary file 1 [file ijms-26-10979-s001.zip › Supplementary Figures.pdf]

International Journal of
Molecular Sciences

MDPI

Supplementary Materials

Flow Cytometric Challenges in Plasmacytoid Dendritic Cell (pDC) Identification: Limitation of BDCA4 (CD304)-Based Gating

Sarolta Demeter [1,2], Tünde Fekete [1], Beáta Scholtz [3], Zoltán Veréb [4], Lajos Kemény [4], Attila Bácsi [1] and Kitti Pázmándi [1,*]

[1] Department of Immunology, Faculty of Medicine, University of Debrecen, H-4032 Debrecen, Hungary; demeter.sarolta@med.unideb.hu (S.D.); fekete.tunde@med.unideb.hu (T.F.); etele@med.unideb.hu (A.B.)
[2] Doctoral School of Molecular Cell and Immune Biology, University of Debrecen, H-4032 Debrecen, Hungary
[3] Department of Biochemistry and Molecular Biology, Faculty of Medicine, University of Debrecen, H-4032 Debrecen, Hungary; scholtz@med.unideb.hu
[4] Regenerative Medicine and Cellular Pharmacology Laboratory, Department of Dermatology and Allergology, Faculty of Medicine, University of Szeged, H-6720 Szeged, Hungary; vereb.zoltan@med.u-szeged.hu (Z.V.); kemeny.lajos@med.u-szeged.hu (L.K.)
* Correspondence: pazmandi.kitti@med.unideb.hu

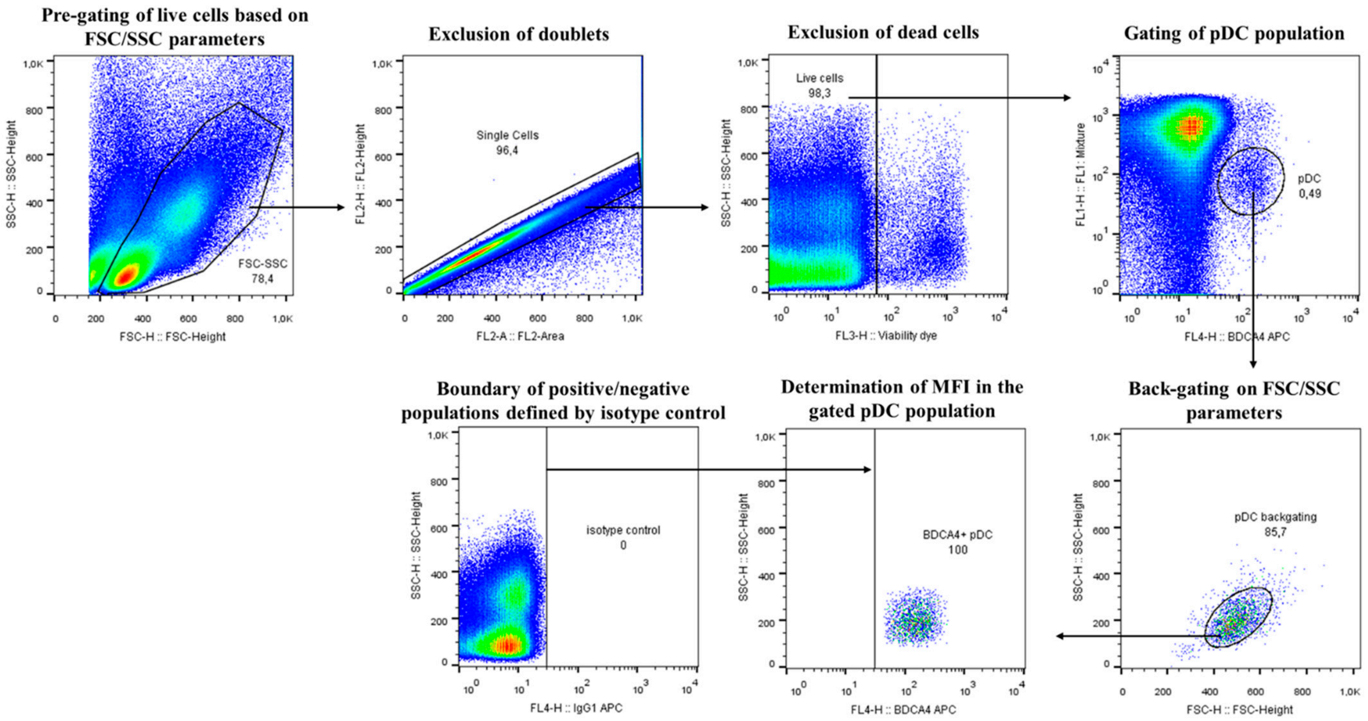

Supplementary Figure S1. Gating strategy of primary human pDCs in PBMCs for flow cytometric analysis. First, live cells were pre-gated based on FSC and SSC parameters, followed by exclusion of doublets and 7-AAD–positive cells. pDCs were then identified as BDCA4$^+$ and antibody cocktail (Mixture)–negative cells. Subsequently, the distribution of cells within the "pDC gate" was analyzed by back-gating on the light scatter parameters to exclude non-pDCs. Cells within the FSC range of 400–600 were defined as pDCs. The percentage of the gated pDC population, as well as the median fluorescence intensity (MFI) of BDCA-4 within the pDC gate, were determined. The threshold of BDCA-4 positivity in the pDC population was set according to the isotype control gate. Representative dot plots are shown.

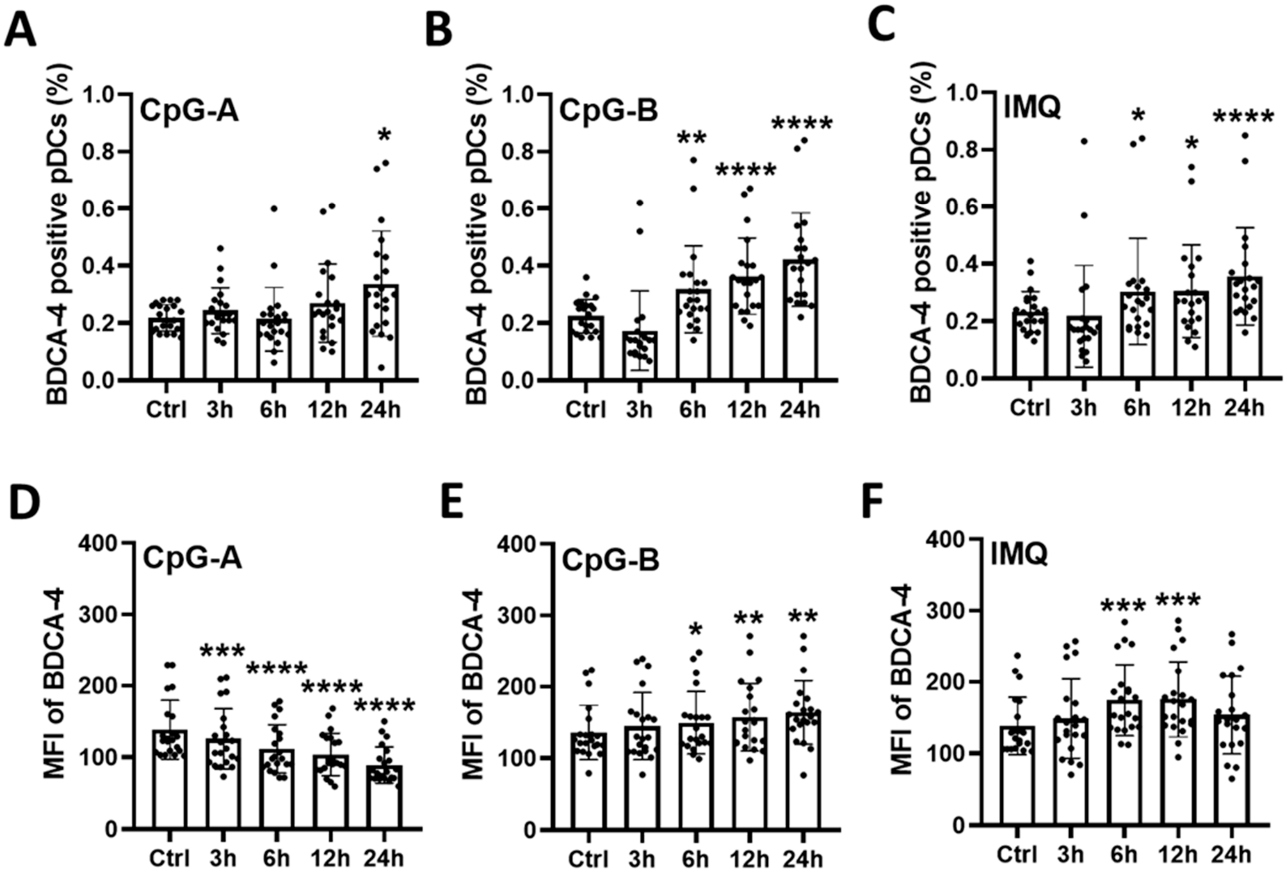

Supplementary Figure S2. Activation-induced alterations in BDCA-4 expression on pDCs. PBMCs were separated from peripheral blood of healthy donors, then the cells were activated with 1 µM CpG-A or 1 µM CpG-B or 5 µg/ml IMQ for 3, 6, 12 or 24 hours. Cells were stained for BDCA-4 and with an antibody cocktail containing non-pDC markers, and the pDC population was identified as BDCA-4 positive but negative for the antibody cocktail by flow cytometry (A-F). The percentage of the gated pDC population (A–C), as well as the median fluorescence intensity (MFI) of BDCA-4 within the pDC gate, were determined (D–F). Data are represented as means ± SD of 21 individual experiments (A-F) and analyzed using one-way ANOVA followed by Bonferroni's post-hoc test. *p < 0.05; **p < 0.01***p < 0.001; ****p < 0.0001 vs control (ctrl). IMQ: imiquimod; MFI: median fluorescence intensity.

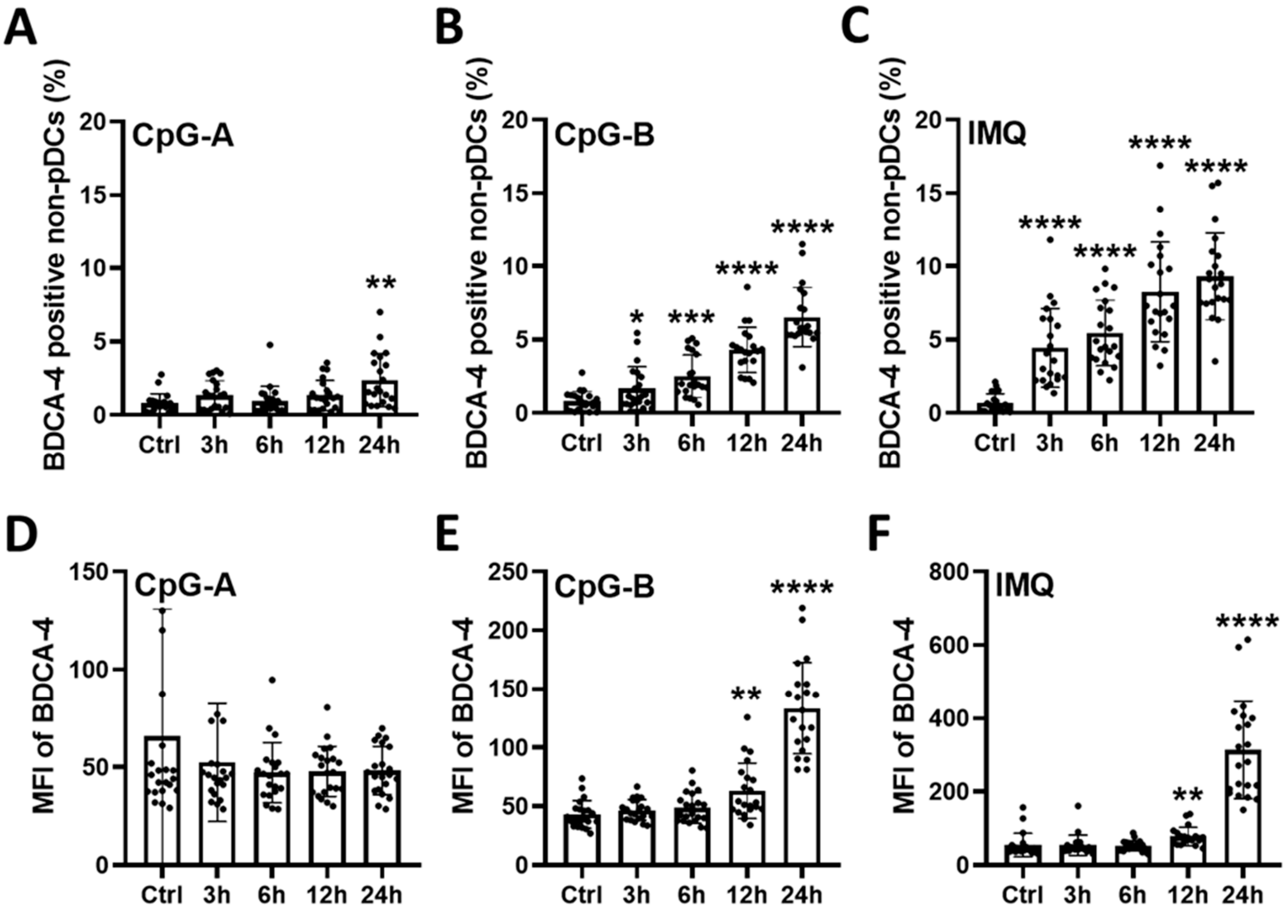

Supplementary Figure S3. Characterization of activation-induced BDCA-4 expression on the non-pDCs population in PBMCs. PBMCs were separated from peripheral blood of healthy donors, then the cells were activated with 1 μM CpG-A or 1 μM CpG-B or 5 μg/ml IMQ for 3, 6, 12 or 24 hours. Cells were stained for BDCA-4 and with an antibody cocktail containing non-pDC markers. The BDCA-4 positive non-pDC population was then identified by flow cytometry (A–F) as cells positive for both BDCA-4 and the non-pDC antibody cocktail. The percentage of the gated BDCA-4 positive non-pDC population (A–C), as well as the median fluorescence intensity (MFI) of BDCA-4 within this population, were determined (D–F). Data are presented as means ± SD of 21 individual experiments (A–F) and were analyzed using one-way ANOVA followed by Bonferroni's post-hoc test. *$p < 0.05$; **$p < 0.01$***$p < 0.001$; ****$p < 0.0001$ vs control (ctrl). IMQ: imiquimod; MFI: median fluorescence intensity.

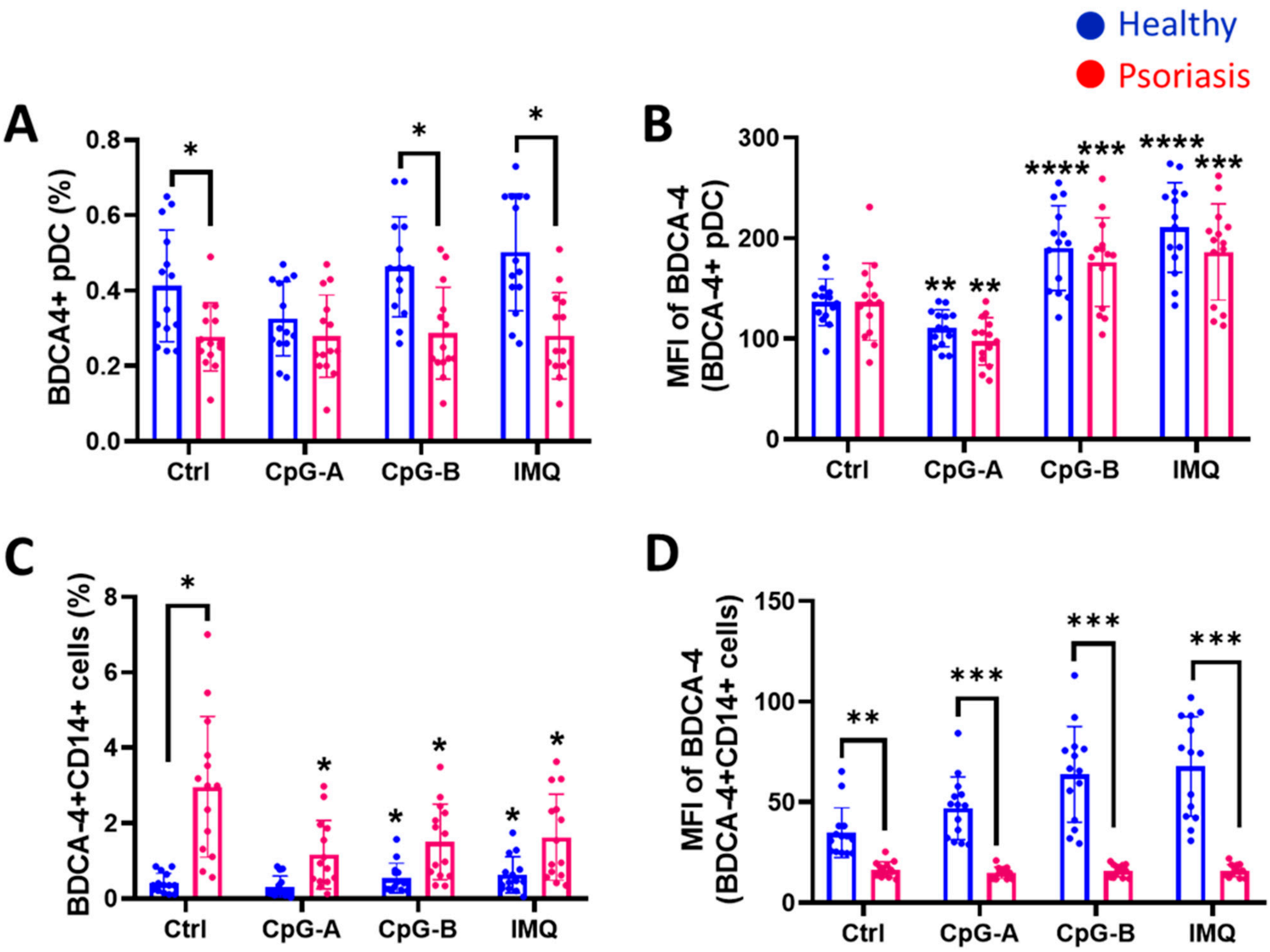

Supplementary Figure S4. Characterization of BDCA-4 positive pDC and non-pDC populations in patients with psoriasis. Peripheral blood samples were collected from healthy volunteers and psoriasis patients. PBMCs were isolated using Ficoll-Paque gradient centrifugation and then activated with 1 μM CpG-A, 1 μM CpG-B, or 5 μg/ml IMQ for 3 hours. After activation, cells were stained for BDCA-4 and CD14 to distinguish the BDCA-4 positive pDC population from the BDCA-4 positive non-pDC population by flow cytometry (A–D). The pDC population was defined as cells positive for BDCA-4 but negative for CD14 (A–B), whereas non-pDCs were defined as cells positive for both BDCA-4 and CD14 (C–D). The percentage of the gated populations (A, C), as well as the median fluorescence intensity (MFI) of BDCA-4 within these populations (B, D), were determined. Data are presented as means ± SD of 14 individual experiments (A–D) and analyzed using one-way ANOVA followed by Bonferroni's post-hoc test. *p < 0.05, **p < 0.01, ***p < 0.001, ****p < 0.0001 vs. healthy or psoriasis control (ctrl). Comparisons not made to the control are indicated separately in the figures. IMQ: imiquimod.

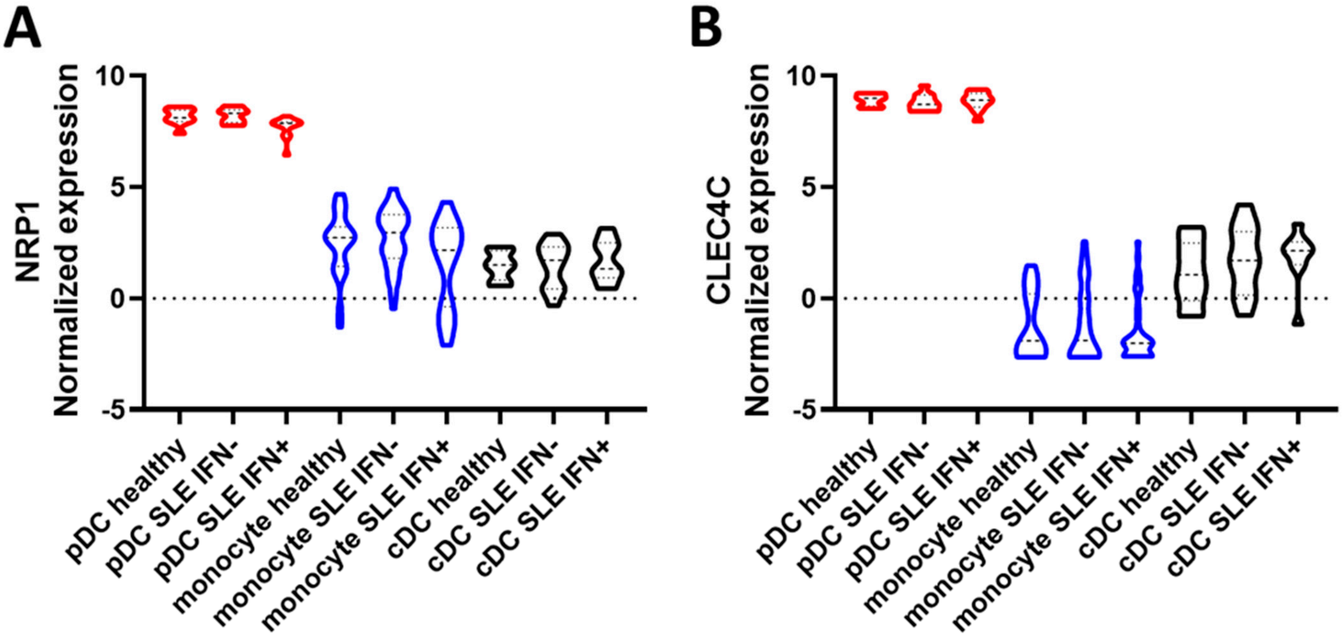

Supplementary Figure S5. Gene expression analysis in SLE patient samples. To characterize the expression of *NRP1* (A) and *CLEC4C* (B) in samples of SLE patients, we downloaded the raw RNA-seq counts of the GSE149050 study [1] were analyzed using the EdgeR program (A-B). Normalized gene expression produced by the program is shown in the graphs, for the following samples: pDC from healthy donors (n=9), and from SLE patients without IFN signature (n=11) or with IFN signature (n=11); monocytes from healthy donors (n=23), and from SLE patients without IFN signature (n=54) or with IFN signature (n=39); cDC from healthy donors (n=10), and from SLE patients without IFN signature (n=11) or with IFN signature (n=9). Pairwise comparisons of cells isolated from healthy controls, either with cells from SLE patients showing an IFN response signature (”IFN+”), or with SLE patients without IFN response signature (”IFN-”) detected no statistically significant differences in gene expression for NRP1 or CLEC4C.
